# Spectrum of Congenital Anomalies among Surgical Patients at a Tertiary Care Centre over 4 Years

**DOI:** 10.1155/2017/4174573

**Published:** 2017-02-09

**Authors:** Arushi Agarwal, K. N. Rattan, Ankur Dhiman, Ananta Rattan

**Affiliations:** Department of Pediatric Surgery, Pt. B.D. Sharma, PGIMS, Rohtak, Haryana, India

## Abstract

*Introduction.* Congenital anomalies are important causes of childhood death, chronic illness, and disability in many countries. Congenital malformations are rapidly emerging as one of the major worldwide problems.* Aim.* To study the percentage of various congenital anomalies among the patients admitted in Department of Pediatric Surgery at a tertiary care centre over a period of four years from 2011 to 2015 in our centre.* Results.* Neural tube defects were found to be the most common anomalies in 24.3% of the children admitted. Other common anomalies were anorectal malformation (20.7%), tracheoesophageal fistula (20%), and intestinal obstruction (14.84%). Majority (60.5%) of the patients were males.* Conclusion.* Congenital malformations are rapidly emerging as one of the major worldwide problems as they can result in long-term disability, which may have significant impacts on individuals, families, health-care systems, and societies. Regular antenatal visits and prenatal diagnosis are recommended for prevention, early intervention, and even planned termination, when needed.

## 1. Introduction

Congenital anomalies are important causes of childhood death, chronic illness, and disability in many countries. Congenital anomalies are also known as birth defects, congenital disorders, or congenital malformations [1]. According to WHO factsheet on 2000–2013 child causes of death, every year, around 276,000 babies die within 4 weeks of birth, worldwide, from congenital anomalies [2]. Congenital anomalies can be defined as structural or functional anomalies (e.g., metabolic disorders) that occur during intrauterine life and can be identified prenatally, at birth, or later in life. Birth defects may be the result of genetic or environmental factors which include errors of morphogenesis, infection, epigenetic modifications on a parental germline, or a chromosomal abnormality. The outcome of the disorder will depend on complex interactions between the prenatal deficit and the postnatal environment [3]. Congenital anomalies can result in long-term disability, which may have significant impacts on individuals, families, health-care systems, and societies. The outcome of children with congenital anomalies in developing countries is worse than in developed countries due to lack of appropriate resources for their management. Congenital anomalies account for 8–15% of perinatal deaths and 13–16% of neonatal deaths in India [4]. As other causes of infant mortality like infections and nutritional deficiencies are being brought under control, congenital malformations are rapidly emerging as one of the major worldwide problems [5, 6]. The prevalence rate of congenital anomalies is increasing due to exposure of teratogens of various kinds [7].

The present study was carried out with an aim to study the percentage of various congenital anomalies among the patients admitted over a period of four years from 2011 to 2015 in our centre. According to English literature this is the first such study of North India.

## 2. Material and Methods

A retrospective analysis was conducted in Department of Pediatric Surgery at Pt. B.D. Sharma, Post Graduate Institute of Medical Sciences, Rohtak, Haryana, from July 2011 to June 2015. The study population comprised 1374 patients admitted with us with congenital anomalies. Relevant information regarding age, sex, birth weight, birth order, and consanguinity was documented. Significant antenatal history like maternal illness, ingestion of drugs, exposure to radiation, mode of delivery, and complications of labor was recorded. Antenatal ultrasonography (USG) findings were noted. Relevant radiological and histohematological tests were carried out. Computed tomography (CT) scan was advised only for certain special cases. The major malformations were divided into central nervous system (CNS), gastrointestinal (GIT), genitourinary (GU), and miscellaneous disorders. Comparison between percentage of affected males and females was made. Many patients with isolated anomalies of lip, palate (like cleft lip/palate), limb deformity (CTEV, syndactyly, polydactyly, etc.), chest deformity (like pectus excavatum), and ear anomalies (microtia, anotia, etc.) were excluded from our study as they did not require immediate surgery and hence were not admitted and managed on OPD basis. Patients who presented in casualty in terminal stage were also not admitted as they could not be operated on and hence were not included in our study. Patients with anomalies of cardiovascular system were referred to pediatric cardiologist and were not admitted with us as there is no pediatric cardiologist at our centre. Hence they were also excluded from this study.

## 3. Results

During the study period 1374 patients were admitted with us with congenital anomalies. Various congenital anomalies were classified according to the system affected (Table 1).

**Figure 1:**
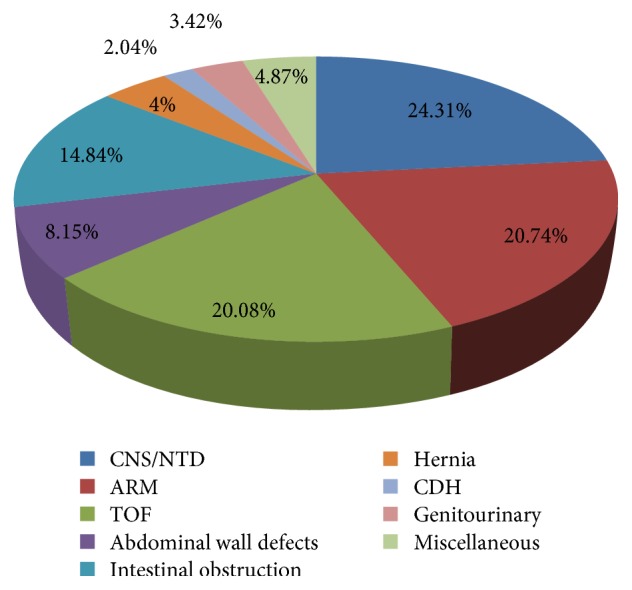
Percentage of children with various congenital anomalies.

Percentage of various anomalies was calculated and compared as shown in Figure 1. NTD was found to be the most common anomaly in 24.3% of the children admitted. It included meningomyelocele, encephalocele, and hydrocephalus. Lumbosacral meningomyelocele was most common anomaly among them.

Next most common anomaly was ARM in 20.74% patients followed by TOF in 20.08% of the patients.

Intestinal obstruction was found to be a major anomaly in this study, occurring among 14.84% patients. It included intestinal atresia in most cases. Others were malrotation of gut, duplication cyst, hypertrophic pyloric stenosis, Hirschsprung disease, meconium plug, and, in few cases, intussusception.

Abdominal wall defects like gastroschisis, omphalocele, patent vitellointestinal duct, exstrophy bladder, and cloacal exstrophy accounted for 8.15% of the anomalies. Inguinal, lumbar, and umbilical hernia were found in 4.44% of cases.

Genitourinary anomalies were found in 3.42%. They comprised posterior urethral valve, pelvic ureteral junction obstruction, and multicystic kidneys.

Congenital diaphragmatic hernia (CDH) was seen in 2.04% of cases. Other anomalies seen in few cases were teratoma, hemangioma, cystic hygroma, ranula tongue, diencephaly, ectopia cordis, and parasitic twinning.

**Figure 2:**
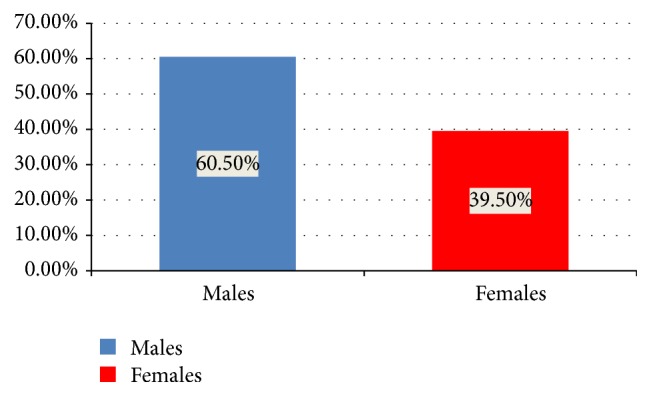
Comparison between number of males and females with congenital anomalies.

Out of the total 1374 patients in our study group, 831 (60.5%) were males while only 543 (39.5%) of them were females.  Figure 2 shows comparison between number of males and females with congenital anomalies.

## 4. Discussion

Congenital malformations are rapidly emerging as one of the major worldwide problems as they can result in long-term disability, which may have significant impacts on individuals, families, health-care systems, and societies. Since the ancient times, congenital anomalies have been topic of frequent discussion and research. The exact causes of malformations remain unknown in a large number of the cases. According to ancient beliefs of negative or supernatural forces, birth defects were a result of divine punishment for wickedness. Over the years, numerous studies have been carried out to determine the prevalence, patterns, possible causations, and other factors of congenital malformations. However despite massive advancements, the magnitude of the problem still to this day causes significant health impacts.

In this study we found that the most common anomalies were NTD (24.3%) followed by ARM (20.7%), followed by TOF (20%), and that males were affected more than females. According to the English literature, there is no study of this kind in North India. Hence this is the first one. Some of the previous studies, performed at other regions, have results similar to our present study. In a study in Dhaka, by Fazle Mubarak Bari [8], GIT accounted for majority of the cases 27% followed by nervous system, 15.7%. 52.8% of the patients were males. According to study by Taksande et al. [4], cardiovascular malformations were most common. 62% males and 38% females were affected in this study which is almost the same as in ours. Sarkar et al. [9] found that the predominant system involved was musculoskeletal system (33.2%) followed by gastrointestinal (GI) system (15%) and central nervous system (CNS) (11.2%) congenital anomalies affected significantly higher proportion of male babies than their female counterparts. Chaturvedi and Banerjee [10] studied rural population of Maharashtra and found that the most common system involved was musculoskeletal (23.65%), followed by CNS (16.12%) and GIT (13.97%). Basavanthappa et al. [11] found that musculoskeletal malformations were the commonest malformation and accounted for 27.5% of all the malformations in a hospital of South India. This was followed by cutaneous 19.16%, genitourinary 15.83%, gastrointestinal 12.5%, neurological 10%, and cardiac malformations 5.83%. in a study in West Bengal, by Pal et al. [12], cardiovascular, musculoskeletal, and genitourinary system were found to be most commonly involved. 62% males and 38% females were affected in this study too.

Despite the high risk of recurrence of congenital malformations, there are no well accepted preventive measures in developing countries like India. It indicates that strong preventive measures for congenital anomalies are needed. Increasing awareness about maternal care during pregnancy, educational programs on congenital malformations and the consequences of consanguineous marriages need to be highlighted to decrease the incidence of congenital anomalies and their comorbidities. Nutritional status of women needs to be improved which includes improving their general nutrition; ensuring adequate intake of specific micronutrients including folic acid, iodine, and iron; and removing harmful substances from the diet, especially alcohol, which may damage the developing embryo or fetus. The periconception period (three months before and after conception) can be targeted by folic acid supplementation [13]. Studies suggest that 70% of neural tube defects can be prevented by the intake of daily dose of 400 *μ*g synthetic folic acid for women of childbearing age [14]. Hence, regular antenatal visits and prenatal diagnosis are recommended for prevention, early intervention, and even planned termination, when needed.

## 5. Conclusion

In this study conducted at our centre, we found that NTD and GI anomalies are very common among those admitted for surgery and that males are affected much more than females.

## Figures and Tables

**Table 1:** Total number of cases in each group of congenital anomalies.

S. number	Anomaly	Total cases	Percentage
1	CNS/NTD	334	24.31%
2	Anorectal malformation (ARM)	285	20.74%
3	Tracheoesophageal fistula (TOF)	276	20.08%
4	Neonatal intestinal obstruction	204	14.84%
5	Abdominal wall defects	112	8.15%
6	Hernia	61	4.44%
7	Genitourinary anomalies	47	3.42%
8	Congenital diaphragmatic hernia (CDH)	28	2.04%
9	Miscellaneous	27	1.96%
